# Othering and Deprioritizing Older Adults’ Lives: Ageist Discourses During the COVID-19 Pandemic

**DOI:** 10.5964/ejop.v16i4.4127

**Published:** 2020-11-27

**Authors:** Roger Andre Søraa, Federico Manzi, Mark W. Kharas, Antonella Marchetti, Davide Massaro, Giuseppe Riva, J. Artur Serrano

**Affiliations:** a Department of Neuromedicine and Movement Science, Faculty of Medicine and Health Sciences, NTNU Norwegian University of Science and Technology, Trondheim, Norway; b Department of Interdisciplinary Studies of Culture, Faculty of Humanities, NTNU Norwegian University of Science and Technology, Trondheim, Norway; c Research Unit on Theory of Mind, Department of Psychology, Catholic University of the Sacred Heart, Milan, Italy; d Humane Technology Laboratory, Catholic University of the Sacred Heart, Milan, Italy; e Applied Technology for NeuroPsychology Laboratory, Italian Institute for Auxology, Milan, Italy

**Keywords:** coronavirus, COVID-19, pandemic, older adults, ageism, othering, deprioritizing

## Abstract

The COVID-19 pandemic is showing troubling othering demographic discourses. For older adults in particular, there are concerning thematics that should be shined light on. In this editorial, we provide perspectives from three countries: Norway, Italy and the United States. We provide four topics of discussion that can be utilized to further understand othering discoures of the COVID-19 pandemic, as well as potential future disasters.

## Older Adults and the COVID-19 Pandemic

Times of crises show what and who are prioritized and deprioritized. Refugee-crises are colored by racism, and homophobia hindered initial responses to HIV/AIDS. Similarly, Fraser et al. (2020), ask what society’s ageist response to the COVID-19 pandemic says about us, and we argue that the initial response paints a grim picture of how we value the lives of older adults. In this editorial, we explore a discourse on the topic of “COVID-19 as an elderly disease”—which was quite prominent in the beginning of the pandemic in multiple countries (Fraser et al., 2020). The deprioritized rhetoric of “only old people die” resulted from a “public discourse [that] presented [COVID-19] as only dangerous to older adults” (Fraser et al., 2020, p. 693), which we explore as an othering of older adults’ lives.

Since its modest beginning on December 31, 2019, COVID-19 has killed more than a million, infected millions, and upended the lives of billions of people in almost every country on the planet. It has also caused the greatest economic collapse since the Great Depression in the 1930s. Several studies found that the effects of COVID-19 symptomatology among older adults are more severe, with increased mortality (Armitage & Nellums, 2020; Fraser et al., 2020). Lockdown policies have increased the morbidity of COVID-19 associated with affective disorders effects due to the isolation of older adults (Armitage & Nellums, 2020; Cavalera et al., 2017). Residents of long-term care facilities are also especially vulnerable (Comas-Herrera et al., 2020). This considerable difference in the mortality rate would create an expectation that special attention and care should be focused on this age group (Comas-Herrera et al., 2020). However, this did not seem to be the case, and public mentions were made in the opposite direction of deprioritizing care for older adults as described in this article.

This can be understood as an implicit geronticide of the world’s older adults population. Brogden (2001, p. 12) describes how geronticide—“the killing of the elderly”—can be understood not in the sensational evil act of slaughtering, but in the silent societal infrastructures that “determine that older people should lose their fundamental right to life” by rationing health resources in favor of other age cohorts. There are troubling cases—most notably in Italy (Vergano et al., 2020) and Spain (de Frutos & Moynihan, 2020; Sills & Lombrana, 2020)—of doctors having to choose who lives and who dies based on the availability of ventilators and other medical equipment.

## The Effect of COVID-19 on Psychological Well-Being of Older Adults

The psychological effects of COVID-19 on the elderly population have been widely recognized in recent months (Armitage & Nellums, 2020; Nobles et al., 2020; Troutman-Jordan & Kazemi, 2020), as well as the importance of analyzing how different countries managed the first months of the pandemic to avoid even worse consequences for older adults (Chaurasia et al., 2020; Monahan et al., 2020). As matter of fact, COVID-19 mainly affects the most physically frail people and those with previous diseases, so older adults are the demographic most at risk (Liu et al., 2020; Shahid et al., 2020). Furthermore, in the early stages of the pandemic, both official communications from several countries first affected by the infection and the media repeatedly stressed that the virus was *primarily* dangerous for the older adults (Ayalon et al., 2020; Jimenez-Sotomayor et al., 2020; Monahan et al., 2020; Rahman & Jahan, 2020).

In the light of this global situation, several studies have addressed the psychological well-being of older adults. In a study conducted in China, data were collected on 1,556 subjects aged between 60 and 80 years (Meng et al., 2020). The results showed that older people displayed symptoms of depression and anxiety during the pandemic. These data are supported by another study by Qiu and colleagues (2020), also carried out in China on 52,730 subjects, which showed that individuals over 60 were more inclined to symptoms including anxiety, depression, specific phobia and compulsive behaviour. More specifically, the high levels of stress experienced by the older adults in this study could be due to evidence that the highest mortality of the virus occurred among older adults (Qiu et al., 2020).

These two studies highlight how older people are susceptible to greater psychological stress, although the study by Qiu and colleagues (2020) also showed that other groups, such as young people and migrant workers, are also particularly vulnerable to psychological stress. A study by López and colleagues (2020) on a Spanish sample of 878 subjects showed that people between 60–70 and 71–80 did not significant differ with respect to the negative effects of COVID-19 on psychological well-being. Although no significant age differences were found, other variables, such as the loss of a loved one during the pandemic, had negative consequences on the psychological well-being of older adults. Finally, another study by Kivi and colleagues (2020) on 1,071 Swedish older adults between 65 and 71 years highlighted how subjective experiences had a significant impact on the psychological well-being of older adults during the pandemic. More specifically, those who worried more about the health status and financial consequences of the pandemic reported lower psychological well-being. However, it is important to point out that these data relate to the first weeks of the pandemic, so further studies need to be conducted to confirm the findings.

These early studies, on the one hand, showed that the psychological well-being of older adults was generally under considerable stress and, on the other, highlighted the importance of considering individual differences to understand the negative psychological effects of the pandemic (Inghilleri et al, 2015; Marchetti et al., 2020).

## The “Othering” of Older Adults

The othering process represents a strongly interdisciplinary concept that intersects different disciplines including psychology, sociology, anthropology, philosophy to name but a few. In this paragraph, despite the complex articulation of this concept, two fundamental perspectives will be presented that highlight two aspects of this process: psychoanalysis, which states that the process of the othering is fundamental for the constitution of the Self, and sociology, where this process is susceptible to historical-cultural changes and often leads to increased inequalities towards different members of society.

From a psychoanalytic perspective, one of the fundamental processes for the construction of the Self is the gradual recognition of the external object, the Other, as an independent subject (Kohut, 1971). Moreover, psychoanalytic theories have shown that We project the undesirable or repressed contents of our unconscious onto the Other (Freud, 1922). This process of creating the Other is, therefore, constitutive of our development (Stern, 1983) and constantly active throughout our lives. This individual process is also found in the social dynamics of the Other (de Beauvoir, 2011) which are subject to historical-cultural changes. Sociology refers to the othering as a negative process that leads to discrimination and exclusion of individuals on the basis of their belonging to marginalized groups of society (Boréus, 2006; Riggins, 1997). This process is even more evident when society is facing moments of severe structural crisis, as is happening during the COVID-19 pandemic.

Therefore, while for psychoanalysis the othering is conceptualized as a fundamental process for the creation of the Self and, consequently, for the individual to be in tune with society, for sociology it represents a process of separation and exclusion of the Other from the society (Hall, 1997). More specifically, the Other "means transforming the Other into another, thus creating a boundary between different and similar, insider and outsider. The emotional and cognitive mechanisms leading to the Other are articulated linguistically and co-constructed interactively but also on a social and supranational level" (Dervin, 2015, p. 2)

Since the 1970s, the process of othering has also been recognized and studied with respect to age, particularly towards the elderly: this process has been defined as *ageism* (Butler, 1969; Nelson, 2002). The World Health Organization (WHO) has defined ageism as "the stereotype, prejudice and discrimination against people based on their age" (WHO, n.d.). Generally, othering—including ageism—has negative effects on both individuals and groups, e.g. through social exclusion and stigmatization. However, in a psychoanalytic discourse, normal development involves construction of the Other, therefore the othering process is common, especially when the Self’s equilibrium is jeopardized. A pandemic certainly jeopardizes society’s equilibrium; therefore, preventing othering on a societal level requires intentional societal action. The ease with which othering occurs is demonstrated by initial public communications regarding the mortality of COVID-19 and, consequently, the rhetoric adopted by the media: a strong tendency to separate Us, the younger and less at-risk people, and the Other, the older people in risk groups. This process seems to have exacerbated stereotypes towards older people, increasing their exclusion from public discourse early on and leading to negative consequences in protecting their physical and mental health (Cesari & Proietti, 2020).

### “Othering” Discourse in Norway, Italy and the USA

For the present analysis of the process of social othering to which older adults have been subjected, three countries were considered: Italy, Norway and the USA. The reason for the inclusion of these countries is a common aspect of the first COVID-19 emergency management: the first official communications were oriented to support the process of othering towards older adults and the more fragile segments of the population, promoting the idea that the virus would only affect older adults and people with previous pathologies—however, as the pandemic progressed, the countries chose widely different approaches to deal with the resulting situations. As will be shown below, the analysis of the official documents and the first news provided by the mass media will show that the process of social othering of older adults is independent from the specific demographic composition of the population in terms of aging. The following examples were chosen based on searches and media examples that emerged during the first couple months after the pandemic outbreak.

In the United States, the Federal Government has issued reminders of the illegality of explicit age-based discrimination in treatment (Fink, 2020). Ethicists have argued for (Emanuel et al., 2020) and against (Bagenstos, 2020) using age as a factor in rationing care. News articles stress the horror of explicitly using age, while recognizing that rationing plans will, implicitly, often result in treatment disparities based on age; there seems to be acceptance if unease (Fink, 2020). However, substantial media coverage of a potentially COVID-related inflammatory condition among young children is noticeable, given that the number of cases—several hundred—is small in the context of the pandemic as a whole (Belluck, 2020). Therefore, while explicit ageism is shunned, implicit bias is alive and well.

Italy's early official communications, as late as February 20, implicitly highlighted the occurrence of COVID-19 only in vulnerable populations (older adults and sick; Italian Ministry of Health, 2020), leading to the Italian media propagating the misleading rhetoric that "only older adults, not the young" are affected and contributing to young people rejecting social distancing restrictions. Furthermore, the spread of the virus among older adults has worsened due to a poor application of preventive measures in nursing homes, also associated with an initial shortage in these contexts of masks and gloves, which has also led to an increase in the infected among employees. Because of this, the media has written about the "massacre of the elderly" in nursing homes (di Fio et al., 2020). This shows some outrage over the de-prioritization of older adults, but an early lack of urgency likely contributed to these situations.

Norway’s border closed down on March 16, when the most restrictive societal regulations since WWII were put in place. But the public discourse was largely around protecting older adults. The Prime Minister specifically recommended that grandparents not undertake childcare while schools were closed (Ripegutu, 2020). There was also a temporary ban on going to cabins, which are often owned by young, healthy people but are located in rural, poorer, and older communities with fewer healthcare resources. This was only put in place to respond to widespread behavior that put older adults at risk, showing that even when government policy tries to foreground older adults’ concerns, societal practice often works against it.

## Four Points of Discussion for Ageism and Othering in Pandemics

Based on these observations we offer the following four provocative questions for stimulating a discussion on what we see as othering of older adults during pandemics—which can be used to further implement more inclusive and just strategies for dealing with pandemics. Being aware of ageist bias in times of crisis can help prepare, mitigate and take action to make societies more inclusive, responsible and just.

### What is Risked by Portraying COVID-19 as an “Elderly-Disease”?

Fraser et al. (2020) have pointed out how this is not a disease of older adults, and that “the effects will be felt by everyone. We all must do our part to curtail its spread” (p. 3). Younger people may chafe at the loss of autonomy or economic pain caused by social distancing, even using #BoomerRemover on social media, while older adults feel forgotten and devalued. How will this affect generational divides, going in both directions?

### What Can Intersections of Social Groups and Empathy Teach Us?

Social determinants of health, especially smoking, homelessness, race, ethnicity, and poverty, play a large role in the morbidity of COVID-19 (Abrams & Szefler, 2020). Addressing these is politically fraught even outside of COVID-19. This time the pandemic especially hit older adults, but would societies have reacted differently if children were the main victims? Articles like Belluck’s (2020) suggest very differently. As Fraser et al. (2020) paradoxically point out: “The death of a young adult merits a life story, while the death of an older adult is too often merely a statistic” (p. 693). The next pandemic might hit different societal groups, which is why it is crucial to learn from othering and ageism, to avoid other future discriminatory discourses.

### Who Decides, Legislates and Governs Who Should Have Access to Healthcare and Resources?

“More than 140 world leaders, experts and elders have made an unprecedented call for guarantees that COVID-19 vaccines, diagnostics, tests and treatments will be provided free of charge to everyone, everywhere” (UNAIDS, 2020). The implementation of such laudable aspirations depends on the actions of the businesses, governments, and hospitals relating to the overall infrastructure of care. It is possible, perhaps probable, that both macro and micro socioeconomic conditions that are emblematic of othering will determine access to care. Widespread recognition and intentional political action will be needed to counteract such disparities.

### How is Governance, Personal Freedom, and Health Balanced in Times of Crises?

One common, if anecdotal, theme is that government communications can contribute to othering discourses and that such discourses exist even when the government works to combat it. These discourses can make it politically or socially difficult to undertake mitigation efforts that curtail personal freedoms and can, when they are implemented, lead to societal backlash against the populations protected by these efforts. Given these realities, how can the government work with other societal influencers to both encourage behavior necessary for public health while not contributing to ageism or other othering?

## Summary

The COVID-19 public discourse, particularly in early stages, led to an othering and deprioritizing of older adults, whose lives were portrayed as less worthy than “the rest” of the population. We problematize this discourse by looking at experiences from the United States, Italy, and Norway. Better inclusion of older adults into the fabrics of societies can make for a more just, caring, and robust world—pre, during and post pandemics. We thus provided four inquiries of ageism and othering that societal stakeholders should consider to avoid othering vulnerable societal groups in future pandemics and disasters.
